# The Parson and His “Blue Ball.”

**Published:** 1902-03

**Authors:** 

**Affiliations:** Lox Box No. 6, Emporia, Va.


### THE PARSON AND HIS "BLUE BALL."

By THE G. U. SPECIALIST,
Lox Box No. 6, Emporia, Va.

I.

An ebon-hued old parson, with gait of sixty years,
Approached my inner office with smiles akin to fears;
His eyes were shining gladly,
The floor he pounded madly,
With feet that flapped *so* sadly,
I lent him both my ears.

II.

A nervous bow he gave me, and with his manner awed me,
As he shifted from foot to foot,
His "umbrerel" it left him, his tile he did divest him,
And then his funeral face began to toot.

III.

Doctor, a "kennel" you will see, sir, is where I place my hand,
It aches and throbs and pains me so, is more than I can stand.
My "grine" is so "techous" and my leg is so sore,
That if I had one on this side it would kill me "shore."

IV.

I caught this "kennel" straining—I works mighty hard,
The people keeps me preachin' to 'em by the yard;
I *exercises* some, too, sir, a walking 'mid my flock,
But that ain't "*nothin'*" compared to *some*,
For I am steady as a clock.

V.

This "kennel" they say is a "*blue ball,*"
Whatever that may be;
But if *all* balls acts like this one,
*Two* balls *is* enough for me.

VI.

Yes, sir; man, this thing "*do*" hurt;
It keeps me "spraddle-legged" and makes me walk so proud and fine,
I fear it may be said
That the old parson's growing young,
When pious I *must* be, and teach my lambs the way to walk
Through all eternity.

VII.

But "shore 'nuff," Boss, iodine *don't* paint, nor "curis intment" either.
I have tried 'em both, so I'm a disbeliever.
You give me something *cooling* like, and take out all this fever,
And when I read my titles clear, we'll all post cross the river,
Where "blue balls" and "kennels" and such mess,
And straining parsons, all,
Will stand erect, ourselves respect,
Without the old "blue ball."

